# Voxel-based ^18^F-FET PET segmentation and automatic clustering of tumor voxels: A significant association with IDH1 mutation status and survival in patients with gliomas

**DOI:** 10.1371/journal.pone.0199379

**Published:** 2018-06-28

**Authors:** Paul Blanc-Durand, Axel Van Der Gucht, Antoine Verger, Karl-Josef Langen, Vincent Dunet, Jocelyne Bloch, Jean-Philippe Brouland, Marie Nicod-Lalonde, Niklaus Schaefer, John O. Prior

**Affiliations:** 1 Department of Nuclear Medicine and Molecular Imaging, Lausanne University Hospital, Lausanne, Switzerland; 2 Department of Nuclear Medicine and Molecular Imaging, Nancy University Hospital, Nancy, France; 3 Department of Nuclear Medicine, University of Aachen, Aachen, Germany; 4 Department of Diagnostic and Interventional Radiology, Lausanne University Hospital, Lausanne, Switzerland; 5 Department of Neurosurgery, Lausanne University Hospital, Lausanne, Switzerland; 6 Department of Pathology and Laboratory Medicine, Lausanne University Hospital, Lausanne, Switzerland; Biomedical Research Foundation, UNITED STATES

## Abstract

**Introduction:**

Aim was to develop a full automatic clustering approach of the time-activity curves (TAC) from dynamic ^18^F-FET PET and evaluate its association with IDH1 mutation status and survival in patients with gliomas.

**Methods:**

Thirty-seven patients (mean age: 45±13 y) with newly diagnosed gliomas and dynamic ^18^F-FET PET before any histopathologic investigation or treatment were retrospectively included. Each dynamic ^18^F-FET PET was realigned to the first image and spatially normalized in the Montreal Neurological Institute template. A tumor mask was semi-automatically generated from Z-score maps. Each brain tumor voxel was clustered in one of the 3 following centroids using dynamic time warping and k-means clustering (centroid #1: slowly increasing slope; centroid #2: rapidly increasing followed by slowly decreasing slope; and centroid #3: rapidly increasing followed by rapidly decreasing slope). The percentage of each dynamic ^18^F-FET TAC within tumors and other conventional ^18^F-FET PET parameters (maximum and mean tumor-to-brain ratios [TBR_max_ and TBR_mean_], time-to-peak [TTP] and slope) was compared between wild-type and IDH1 mutant tumors. Their prognostic value was assessed in terms of progression free-survival (PFS) and overall survival (OS) by Kaplan-Meier estimates.

**Results:**

Twenty patients were IDH1 wild-type and 17 IDH1 mutant. Higher percentage of centroid #1 and centroid #3 within tumors were positively (P = 0.016) and negatively (P = 0.01) correlated with IDH1 mutated status. Also, TBR_max_, TBR_mean_, TTP, and slope discriminated significantly between tumors with and without IDH1 mutation (P range 0.01 to 0.04). Progression occurred in 22 patients (59%) at a median of 13.1 months (7.6–37.6 months) and 13 patients (35%) died from tumor progression. Patients with a percentage of centroid #1 > 90% had a longer survival compared with those with a percentage of centroid #1 < 90% (P = 0.003 for PFS and P = 0.028 for OS). This remained significant after stratification on IDH1 mutation status (P = 0.029 for PFS and P = 0.034 for OS). Compared to other conventional ^18^F-FET PET parameters, TTP and slope were associated with PFS and OS (P range 0.009 to 0.04).

**Conclusions:**

Based on dynamic ^18^F-FET PET acquisition, we developed a full automatic clustering approach of TAC which appears to be a valuable noninvasive diagnostic and prognostic marker in patients with gliomas.

## Introduction

Gliomas constitute the most frequent brain tumors [1] and are heterogeneous in histology, genetics, and outcome [2]. In particular, the prognostic information of mutations in isocitrate dehydrogenase (IDH) 1 and 2 has been described by several studies [3]. IDH1 mutation is a strong and independent predictor of survival [4]. A longer survival is observed in patients with gliomas harboring the presence of IDH1 or IDH2 mutations [5], whereas the absence of IDH1 appears as a strong predictor for poor prognosis [6]. The World Health Organization has recently updated Central Nervous System (CNS) classification by integrating these molecular parameters for diagnostic and prognostic evaluation of gliomas [7].

Compared to ^18^F-Fluorodeoxyglucose (^18^F-FDG) which shows high tracer uptake in normal gray matter, radiolabeled amino acids such as ^11^C-methionine (^11^C-MET), ^18^F-fluoro-L-dopamine (^18^F-FDOPA) and ^18^F-fluoro-ethyl-tyrosine (^18^F-FET PET) [8,9] exhibit a low tracer uptake in the normal brain and can depict brain tumors with a high tumor to background contrast. These tracers are increasingly used in the diagnostic workup of patients with gliomas, including differential diagnosis, evaluation of tumor extension, treatment planning and follow-up [10]. They may add some value either for prognostic or classification purposes [11,12]. The use of amino acid PET was recently recommended by the Response Assessment in Neuro-Oncology (RANO) working group as an additional tool for evaluating gliomas [13]. As both ^18^F-FDOPA and ^18^F-FET are not integrated into any metabolic pathway, FET uptake signal in tumors is mainly due to perfusion and to the expression of its specialized transporter namely L-amino acid transporter (LAT) [14]. In particular, the correlation between IDH mutation status and imaging metabolite remains unclear [15].

Dynamic ^18^F-FET PET showed some interest for tumor grading [16]. Procedures for imaging with ^18^F-FET PET usually consist of a dynamic acquisition of 40 to 50 minutes immediately started after the radiotracer injection. From these acquisitions, time-activity curves (TAC) can be computed based on regions of interest (ROI) such as 2-dimensional (2D) circular ROI, 3-dimensional (3D) spherical ROI of 2mL centered by the maximum standardized uptake value (SUV_max_) [17] or fixed threshold which can change but is classically 90% of SUV_max_ [18]. From these TAC, some features may be extracted such as time-to-peak (TTP), maximum activity, area under curve, shape of curve and can be correlated to clinical outcomes [19]. IDH1 and 2 mutations were more frequent in tumors with homogeneous increasing (90%) and focal decreasing (79%) TAC [20]. The pathophysiological mechanisms of these TAC are not fully known or understood, and may reflect many aspects of tumor microenvironment such as neoangiogenesis, microvessel density, perfusion and tumor phenotype [21–23]. Therefore, the main reason for using TAC is a global integration of multiple factors that could lead to a global tumor phenotype and an individual prognostic based on the shape of the dominant curve [20]. The shape of curve is classically assessed by a visual analysis [16] and may be complemented with objective criteria such as TTP or slope [17,20]. To the best of our knowledge, most of studies have included an analysis of TAC at the tumor level (one TAC for the whole tumor) but not at the voxel level. Then, aim was to develop a full automatic clustering approach of TAC from dynamic ^18^F-FET PET and evaluate its association with IDH1 mutation status and survival in patients with gliomas. Results were secondarily compared to those of other conventional ^18^F-FET PET parameters.

## Materials and methods

### Patients

Between August 2009 and December 2015, a total of 52 patients with suspected primary brain tumor on conventional magnetic resonance imaging (MRI) were retrospectively enrolled in this study. Every patient underwent a ^18^F-FET PET/CT at an initial stage before any planned subsequent surgical stereotaxic tumor biopsy or any treatment (tumor resection, chemotherapy, radiotherapy). Patients who required rapid surgery (<2 weeks) due to mass effect or intracerebral hemorrhage, as well as patients with history of brain biopsy, surgery or brain treatment were excluded. Also, 15 patients with normal and non-segmentable ^18^F-FET PET images by our semi-automatic technique were excluded. All patients underwent imaging procedures as standard care and gave written informed consent before the ^18^F-FET PET/CT. Collection and analysis of data was retrospective and performed after de-identification. The local Ethics Research Committee of the State of Vaud took into account the retrospective analysis of our database, approved the protocol (no. 2017–00758) and waived the requirement for patient informed consent for the study analysis.

### ^18^F-FET PET acquisition

Patients underwent a dynamic ^18^F-FET PET/CT on Discovery D690 time-of-flight (27 patients) and Discovery LS (10 patients) (GE Healthcare, Waukesha, WI, USA). They were required to fast for at least 4 hours before undergoing the planned ^18^F-FET injection as recommended by EANM guidelines [24]. After intravenous injection of 214±25 MBq (range 145–295 MBq) of ^18^F-FET, PET images were acquired using a dynamic protocol over 50 minutes (10 frames of 5 min; 3.3-mm or 4.2-mm section thickness; 24 cm field-of-view, matrix size of 256 × 256). Calibration for the two machines was the same. ^18^F-FET PET images were reconstructed by the iterative method ordered-subset expectation maximization (3 iterations and 16 subsets) including a Gaussian post reconstruction filter of 5 mm in full width at half maximum (FWHM). Raw data were corrected for attenuation by soft-tissue and skull bone using an unenhanced CT brain (120 kV, 10 mAs), and normalized to the injected dose and body mass by calculation of the SUV.

### Dynamic ^18^F-FET PET segmentation

**Fig 1:**
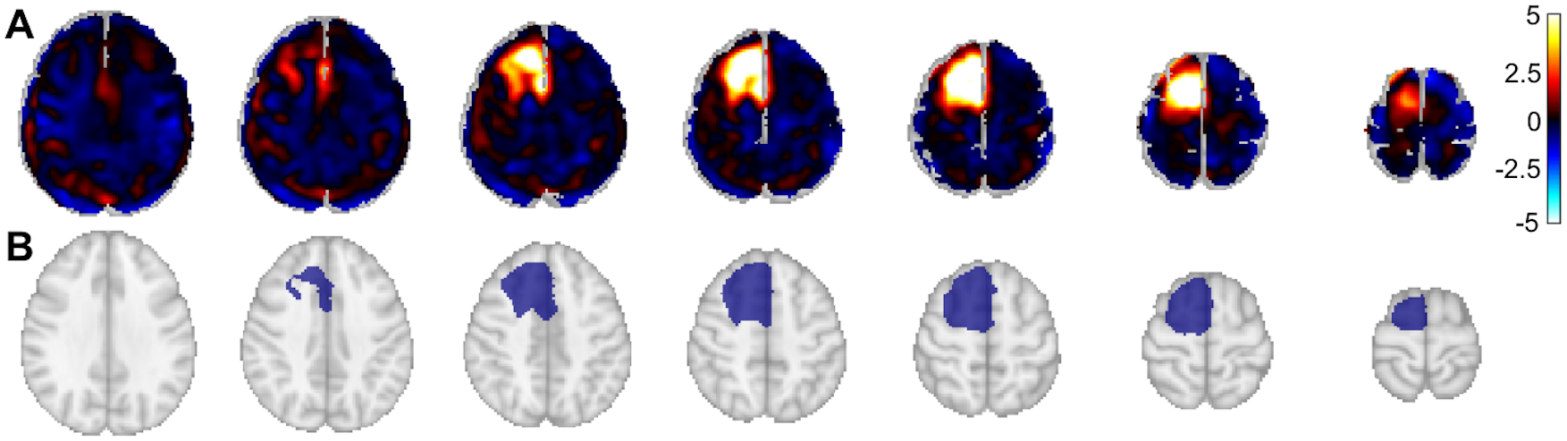
Generation of the mask for each tumor. A. Z-score map obtained from SPM12 between each patient and a population-averaged normal ^18^F-FET PET brain template. B. The mask obtained from connected regions of previous Z-score map, followed by a dilatation to not miss adjacent pertinent voxels.

All dynamic ^18^F-FET PET brain image volumes were temporally realigned to the first dynamic acquisition, coregistered and spatially normalized onto the Montreal Neurological Institute template (McGill University, Montreal, Canada). Dimensions of the resulting voxels were 2x2x2 mm^3^. Images were smoothed using a Gaussian filter (FWHM 8 mm). Preprocessing was performed using the SPM (SMP12) software implemented in Matlab version R2015a (Mathworks Inc., Sherborn, MA). In order to perform a semi-automatic contouring, a mask for glial tumor was obtained using Z-score maps (Fig 1A). Z-score maps were obtained from the difference between each patient static acquisition (which is a summation images from 40 to 50 minutes) and an averaged normal ^18^F-FET PET brain template generated from visually normal ^18^F-FET PET (absence of abnormal ^18^F-FET uptake detected by a trained nuclear physician on static images and t-test parametric image) of 41 patients with untreated gliomas using a Z-score > 2.5 at the voxel level for tumor delineation and k clusters ≥ 250 voxels. Images and masks were visually controlled by 2 expert brain ^18^F-FET PET interpreters in consensus, followed by a dilatation (3x3x3 structure element with a square connectivity equal to one and 2 iterations) to not miss adjacent pertinent voxels (Fig 1B).

### Voxel clustering and extraction of dynamic ^18^F-FET PET TAC

As we were interested in the shape of the TAC which have shown some interest in differentiating IDH mutation status [19,20], the tumor proportion of TAC shape was investigated. A non-supervised approach with k-means clustering to classify each voxel of the tumor mask was used. As recommended [25], we first normalized from the mean and standard deviation dynamic ^18^F-FET PET. Each time series (corresponding to a tumor voxel over the 10-time frames) was Z-normalized to have a mean set to 0 and a standard deviation to 1 using the following formula T = (T-mean(T)) / std(T) where T is the 10-dimensional vector of tumor voxel activity over time. Z-normalization is critical to compare time series. Indeed, as empirically demonstrated by Keogh et al. [26], similarity measure on unnormalized data gives wrong results.

**Fig 2:**
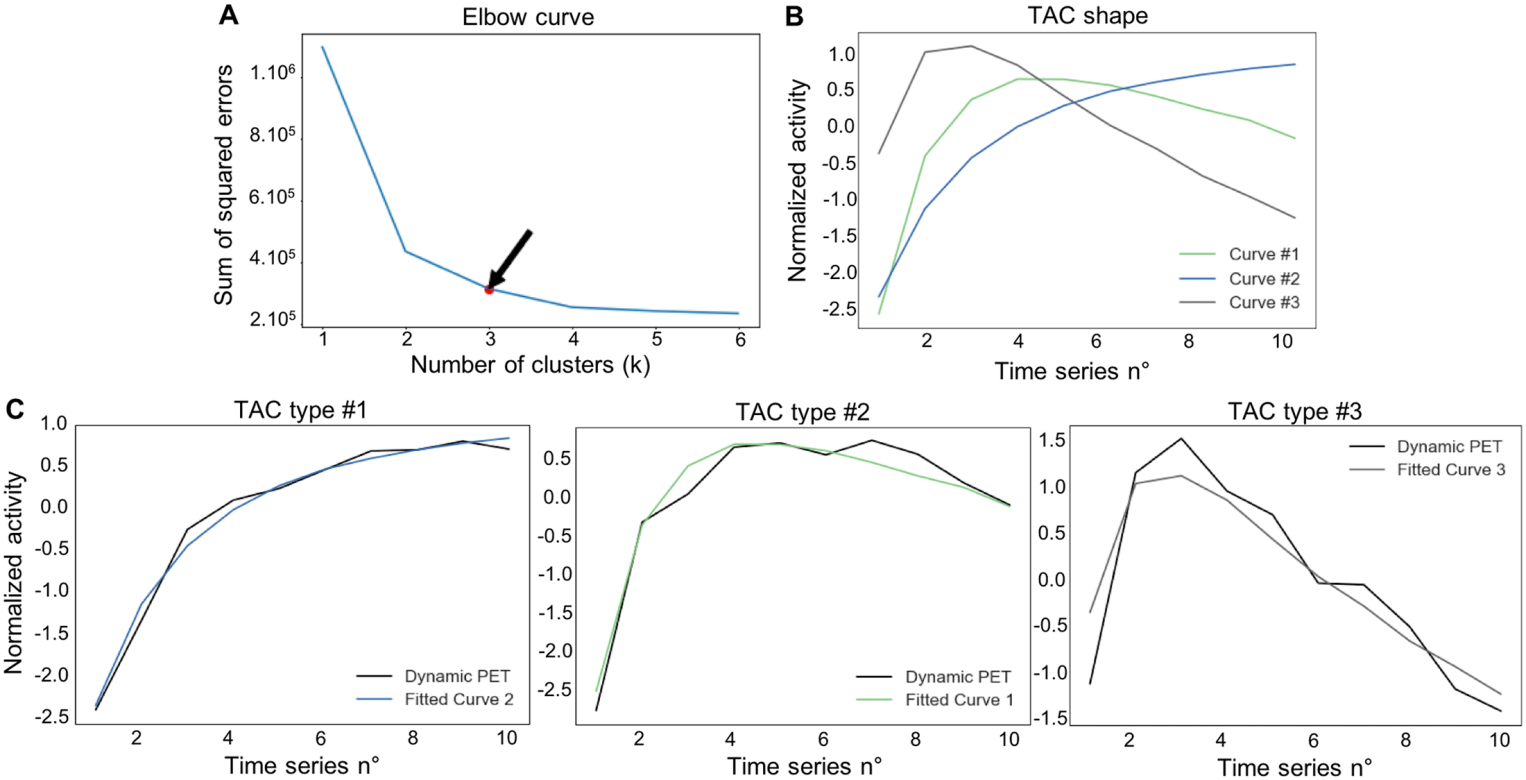
Voxel clustering and features extraction from dynamic ^18^F-FET PET. A. The Elbow method, where the abscissa is the time series number and the ordinate axis the sum of squared errors between each voxel real value and the associated centroid which fitted best the time-activity curve (TAC). Black arrow represents the break point in the curve, corresponding to 3 clusters centroids. B. The 3 centroids included in the final model; centroid #1: slowly increasing slope; centroid #2: increasing slope and slowly decreasing slope; centroid #3: rapidly increasing followed by rapidly decreasing curve. C. Centroid #1, centroid #2 and centroid #3 fitted with 3 TAC (black curves) of voxels.

The number of clusters was fixed a priori and similar time series were clustered together using dynamic time warping (DTW) Euclidian distance. DTW aims to find the optimal non-linear alignment between two-time series. More details about DTW are given in S1 Appendix. K-means algorithm was used for tumor voxel clustering. Therefore, the DTW algorithm with k-means clustering returns the k centroids that maximize intra-cluster similarity and maximize inter-cluster dissimilarity. Each voxel was then classified in one of the k centroids based on the maximization of similarity as measured with Euclidian distance. To find the optimal number of centroids, in which we wanted that each voxel could be classified, the elbow method was used (Fig 2A). The elbow method calculates for each value of k (where k is the number of clusters) the sum of squared errors (SSE) which was obtained by summing the squared error between each original TAC from tumor voxel and the cluster centroids (produced by the DTW and k-mean clustering) from which it was the closest. The idea of the elbow method is to choose the k when the SSE stops decreasing “abruptly”, which produces the so-called "elbow effect" in the graphics of SSE. Indeed, adding any cluster after this would complicate more the model without significantly improving performance as measure with SSE. According to the findings of the elbow method and the well-known different types of tumor TAC which are currently used to differentiate low-grade gliomas from high-grade [20,27,28] a number of 3 was set for k, returning 3 centroids and used as main TAC patterns in the current study (Fig 2B). For convenience reason, we labeled each resultant centroids produced by the DTW algorithm identically as already mentioned in previous papers [27,28] (Fig 2C): centroid #1: slowly increasing slope; centroid #2: rapidly increasing followed by slowly decreasing slope; and centroid #3: rapidly increasing followed by rapidly decreasing slope). Each voxel of patients’ tumors ROI was attributed to one of the 3 cluster centroids from which the TAC of that voxel was the closest. Then, the number and percentage of each type of centroids for each patient was computed. To assess the performance of this analysis, results were compared to those of other conventional ^18^F-FET PET parameters (maximum and mean tumor-to-brain ratios [TBR_max_ and TBR_mean_], time-to-peak [TTP] and slope) between IDH1 wild-type and IDH1 mutant tumors. As previously described TBR were calculated by dividing the SUV_max_ and SUV_mean_ of the tumor by the SUV_mean_ of a larger crescent shape ROI placed in the semioval centre of the contralateral unaffected hemisphere [29,30]. TTP was the time in minutes from the beginning of the dynamic acquisition up to the maximum SUV of the lesion. The slope of the TAC in the late phase of ^18^F-FET uptake was quantified by fitting a linear regression line to the late phase of the curve (20–50 min post-injection) and expressed as SUV/hour [17]. All these computations were performed using python 2.7 with nilearn- and scikit-learn packages [31].

### Histopathology and determination of IDH1 mutation status

Histopathologic classification and tumor grading were performed according to the World Health Organization (WHO) guidelines at the respective time point of histopathologic assessment by trained neuropathologists blinded to MRI and ^18^F-FET PET brain images. All tumors were classified according to the 2007 WHO classification of tumors of the CNS [32]. IDH1 mutation status was analyzed via immunostaining against the common mutant protein IDH1 variant R132H (anti-IDH1 R132H/DIA-H09, mouse monoclonal anti-brain tumor marker; Dianova GmbH, Hamburg, Germany).

### Statistical analysis

Continuous variables were expressed as median with their 25^th^-75^th^ interquartile range. Categorical variables were presented with absolute and relative frequencies. Characteristics of populations were compared by using Student’s t-test or a bilateral Mann Whitney U test for quantitative variables and chi-squared for comparison between categorical variables. Prognostic value of dynamic ^18^F-FET TAC and IDH1 mutation status was assessed in terms of progression free-survival (PFS) and overall survival (OS). PFS was defined as the time between initial ^18^F-FET PET and demonstration of unequivocal tumor progression on follow-up imaging with MRI based on RANO criteria [33] and/or death. OS was defined as time from the baseline ^18^F-FET PET until death from any cause. Patients with no known progression or survival were censored as of their last visit or their last scan date. Survival functions were obtained from Kaplan-Meier estimates and compared using the log-rank test. Using X-tile software version 3.6.1 (Yale University School of Medicine, New Haven, CT) [34], the optimal tumor voxel percentage of each TAC and the optimal TBR_max_, TBR_mean_, TTP, and slope to predict PFS and OS served as cutoff to separate high-risk and low-risk patients. All these statistical analyses were performed using SPSS software (version 23 for Windows 2010, SPSS Inc., Chicago, IL, USA). A *P* value < 0.05 was considered statistically significant.

## Results

### Patient characteristics

Clinical and histopathological data are given in Tables 1 and 2 respectively. Out of the 52 patients included, 37 patients (14 women; mean age: 45±13 y) with positive ^18^F-FET PET and histologically proven brain tumor could be segmented and were retained in the final analysis. Twenty patients were IDH1 wild-type and 17 IDH1 mutant. These subgroups differed in terms of age (P = 0.04) and number of patients treated with radiochemotherapy (P = 0.003).

**Table 1:** Population characteristics.

Characteristics	All patients (*n* = 37)	IDH1 wild-type tumors (*n* = 20)	IDH1 mutant tumors (*n* = 17)	*P* value
Age	44.7 (36;53)	49.1 (40–59)	42.6 (36–45)	0.04
Female gender	14 (37.8)	8 (40)	6 (35.3)	0.77
Delay between ^18^F-FET PET and histopathological diagnosis*	1.1 (0.5;1.7)	0.8 (0.2–1.3)	1.4 (1.0–2.1)	0.49
Treatments				
Surgery	6 (16.2)	1 (5)	5 (29.4)	0.05
Radiochemotherapy	11 (29.7)	10 (50)	1 (5.9)	0.003
Chemotherapy	9 (24.3)	4 (20)	5 (29.4)	0.51
Surgery + radiochemotherapy	8 (21.6)	3 (15)	5 (29.4)	0.65
None	3 (8.1)	2 (10)	1 (5.9)	0.29

Values are median (25^th^-75^th^ interquartile range) or n (%).

*expressed as months

IDH, isocitrate dehydrogenase

**Table 2:** Histopathological and ^18^F-FET PET data.

	Histopathological data				^18^F-FET PET data								
#	Type	MIB-1	IDH1 mutation	WHO grade	Centroid #1** (%)	Centroid #2** (%)	Centroid #3** (%)	SUV_max_ (g/ml)	SUV_mean_ (g/ml)	TBR_max_	TBR_mean_	TTP (min)	Slope (SUV/h)
1	Oligodendroglioma	5	+	II	98.2	0.1	1.7	3.6	2.1	4.1	2.4	45	1.23
2	Anaplastic astrocytoma	20	-	III	5.2	54.6	40.2	2.4	1.5	4.0	2.5	15	-0.72
3	Anaplastic astrocytoma	5	-	III	27.3	70.8	1.9	2.2	1.8	2.4	2.0	25	-0.43
4	Oligodendroglioma	15	+	II	7.5	57.4	35.1	3.4	2.1	3.8	2.3	15	-0.28
5	Low-grade glioma	0	+	I	97.2	0	2.8	1.4	1.1	1.6	1.2	30	0.14
6	Anaplastic oligoastrocytoma	Unknown	+	III	95.3	4.7	0	4.0	2.2	3.1	1.7	45	0.05
7	Secondary glioblastoma*	15	+	IV	100	0	0	1.1	1.0	1.2	1.1	45	0.35
8	Anaplastic oligoastrocytoma	10	+	III	5.2	33.9	60.9	1.7	1.2	2.4	1.7	10	-0.32
9	Primary glioblastoma	30	-	IV	99.9	0	0.1	3.7	2.1	4.4	2.6	45	0.79
10	Anaplastic oligodendroglioma	60	-	III	54.3	45.7	0	2.3	1.6	3.3	2.3	35	-0.26
11	Oligoastrocytoma	15	+	II	71.7	28.3	0	2.2	1.7	2.8	2.1	40	0.11
12	Oligoastrocytoma	15	-	II	10.9	24.8	64.3	2.9	2.1	3.3	2.5	10	-0.83
13	Primary glioblastoma	40	-	IV	27.6	31.3	41.1	2.4	1.6	3.0	2.0	10	-0.61
14	Anaplastic oligoastrocytoma	30	-	III	89.9	9.7	0.4	2.4	1.9	2.0	1.6	45	-0.07
15	Anaplastic oligodendroglioma	60	-	III	15.5	33.5	51	2.5	1.8	3.2	2.1	10	-0.65
16	Oligoastrocytoma	10	-	II	11.8	28.8	59.4	3.3	2.0	4.1	2.5	10	-0.96
17	Oligodendroglioma	10	+	II	14.6	83	2.4	2.6	1.4	2.9	1.6	15	-1.01
18	Anaplastic astrocytoma	10	+	III	12.6	85.7	1.7	2.2	1.7	1.8	1.4	20	0.06
19	Anaplastic astrocytoma	40	-	III	81.9	10.2	7.9	4.0	2.3	6.7	3.8	40	0.55
20	Diffuse astrocytoma	5	-	II	21.1	66.4	12.5	3.3	2.4	3.7	2.6	20	-1.16
21	Ganglioglioma	2	-	II	1.8	28.9	69.3	2.6	1.5	4.3	2.5	10	-0.70
22	Anaplastic oligoastrocytoma	2	+	III	98.6	0.1	1.3	2.2	1.7	2.4	1.9	45	1.11
23	Low-grade glioma	3	-	I	6.5	76.7	16.8	2.6	1.9	2.9	2.1	15	-0.50
24	Anaplastic astrocytoma	10	-	III	14.5	47.6	37.9	2.4	1.6	3.4	2.3	15	-0.68
25	Anaplastic oligodendroglioma	20	+	III	42.2	28.8	29	3.5	2.3	3.9	2.5	20	-0.91
26	Oligodendroglioma	3	+	II	98.8	1.1	0.1	2.7	2.0	3.4	2.5	45	0.34
27	Gemistocytic astrocytoma	0	+	II	93.5	6.5	0	2.2	1.7	2.9	2.2	35	0.32
28	Oligoastrocytoma	1	+	II	100	0	0	1.6	1.4	2.3	2.0	45	0.56
29	Oligoastrocytoma	10	+	II	97.2	2.7	0.1	3.3	2.2	3.4	2.1	45	0.34
30	Primary glioblastoma	30	-	IV	9.3	50.3	40.4	5.4	2.9	5.3	2.8	10	-0.94
31	Oligoastrocytoma	10	-	II	93.5	3.3	3.2	4.5	3.3	4.9	3.8	40	0.43
32	Diffuse astrocytoma	3	-	II	0	0.1	99.9	1.4	1.2	1.9	1.4	5	-0.66
33	SEGA	1	-	I	100	0	0	2.9	1.8	4.1	2.6	45	0.54
34	Oligodendroglioma	5	+	II	92.5	7.5	0	2.2	1.8	2.8	2.3	45	0.13
35	Anaplastic oligoastrocytoma	8	+	III	30.8	57.1	12.1	1.8	1.4	2.6	2.0	25	-0.08
36	Primary glioblastoma	80	-	IV	73.5	26.5	0	1.9	1.4	2.7	2.0	45	-0.31
37	Anaplastic astrocytoma	40	-	III	50.6	22.5	26.9	2.1	1.8	2.2	1.9	20	0.04

IDH, isocitrate dehydrogenase; WHO, World Health Organization; SEGA, subependymal giant cell astrocytoma; SUV, standardized uptake value; TBR, tumor-to-brain ratio; TTP, time-to-peak;

*malignant transformation from oligodendroglioma;

** tumor proportion of each centroid

### Extraction of dynamic ^18^F-FET PET TAC and association with IDH1 mutation status

**Fig 3:**
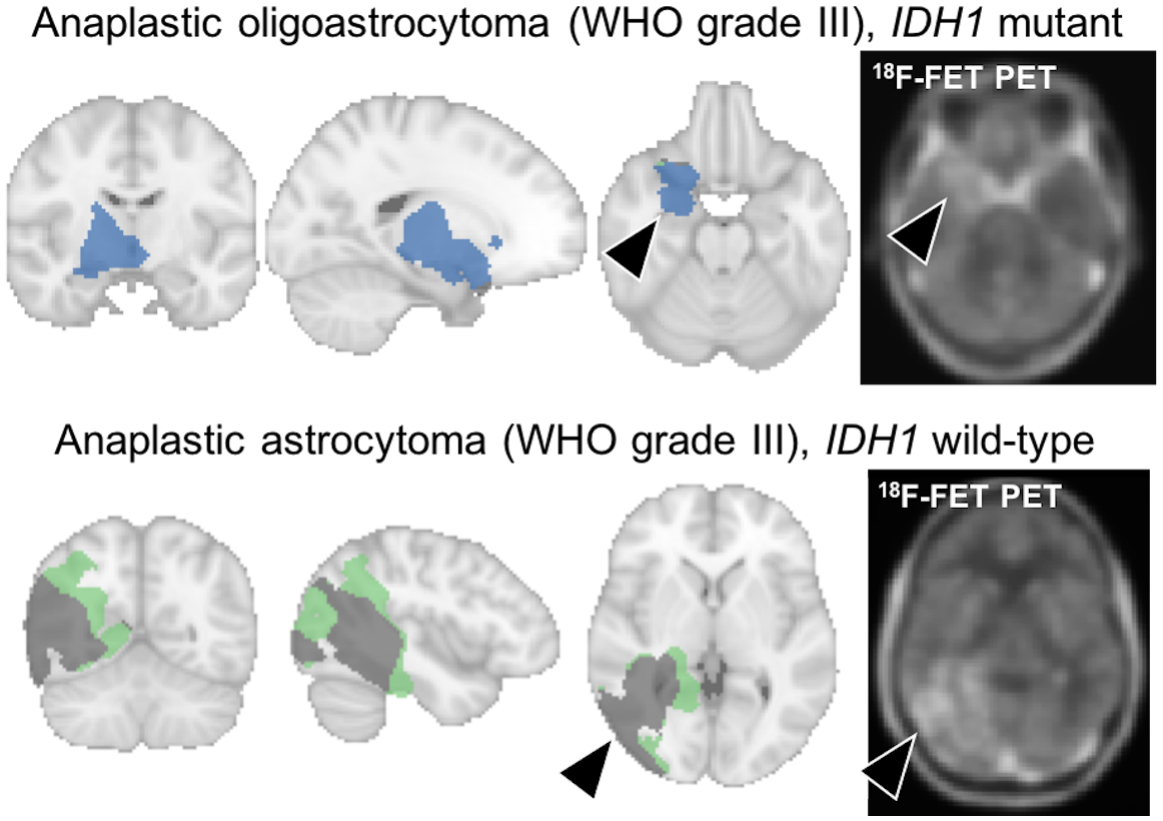
Example of 2 patients with anaplastic oligoastrocytoma IDH1 mutant (patient #22) and anaplastic astrocytoma IDH1 wild-type (patient #24) and their spatial repartition of centroid (blue: Tumor repartition of centroid #1, green: Tumor repartition of centroid #2, grey: Tumor repartition of centroid #3).

**Fig 4:**
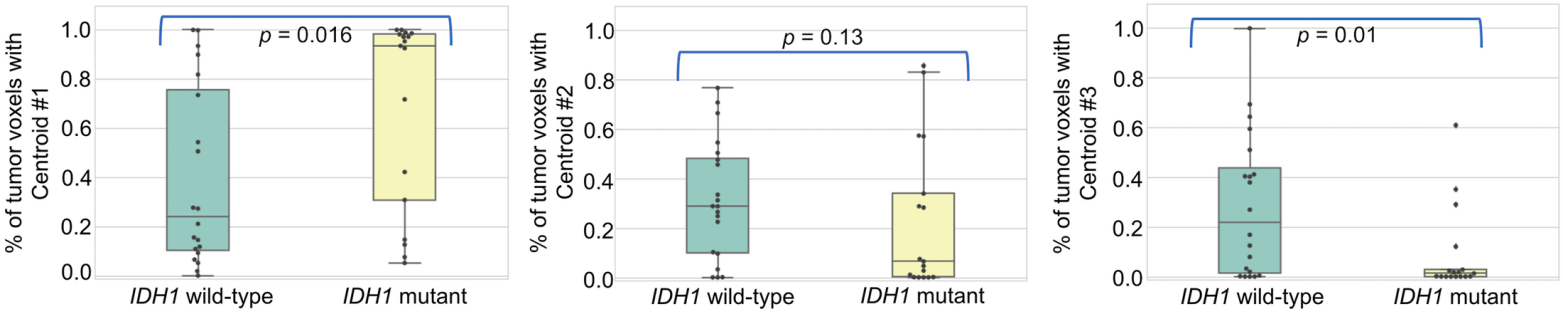
Association with IDH1 mutation status. Boxplots with percentage of tumor voxels with centroids #1, #2 and #3 (from left to right) according to the IDH1 mutation status.

For all tumors, the proportion of each centroid is given in Table 2. Example of 2 patients with anaplastic oligoastrocytoma IDH1 mutant (patient #22) and anaplastic astrocytoma IDH1 wild-type (patient #24) and their spatial repartition of centroid is illustrated in Fig 3. It showed a higher tumor proportion of centroid #1 (98.6%) in IDH1 mutant tumor and a higher tumor proportion of centroid #2 and centroid #3 (47.6% and 37.9% respectively) in IDH1 wild-type tumor. As shown in Fig 4 using boxplots, the automatic voxel clustering based on the 3 fixed centroids confirmed that a higher percentage of tumor voxel with centroid #1 and centroid #3 was positively (P = 0.016) and negatively (P = 0.01) correlated with the IDH1 mutant status respectively. No difference was found with centroid #2 (P = 0.13). As shown in Table 3, TBR_max_, TBR_mean_, TTP, and slope discriminated significantly between IDH1 wild-type and IDH1 mutant tumors (P range 0.01 to 0.04).

**Table 3:** Association with IDH1 mutation status.

	All patients	IDH1 wild-type tumors	IDH1 mutant tumors	*P* value
TBR_max_	2.9 (2.4;3.6)	3.3 (2.7;4.1)	2.6 (2.2;3.3)	0.02
TBR_mean_	2.1 (1.7;2.3)	2.2 (2;2.5)	1.9 (1.7;2.2)	0.02
TTP (min)	25 (15;45)	17.5 (10;40)	40 (20;45)	0.04
Slope (SUV/h)	-0.08 (-0.66;0.34)	-0.56 (-0.7;-0.05)	0.13 (-0.08;0.35)	0.01

Values are median (25^th^;75^th^ interquartile range)

SUV, standard uptake value; TBR, tumor-to-brain ratio; TTP, time-to-peak

### Association with survival

**Fig 5:**
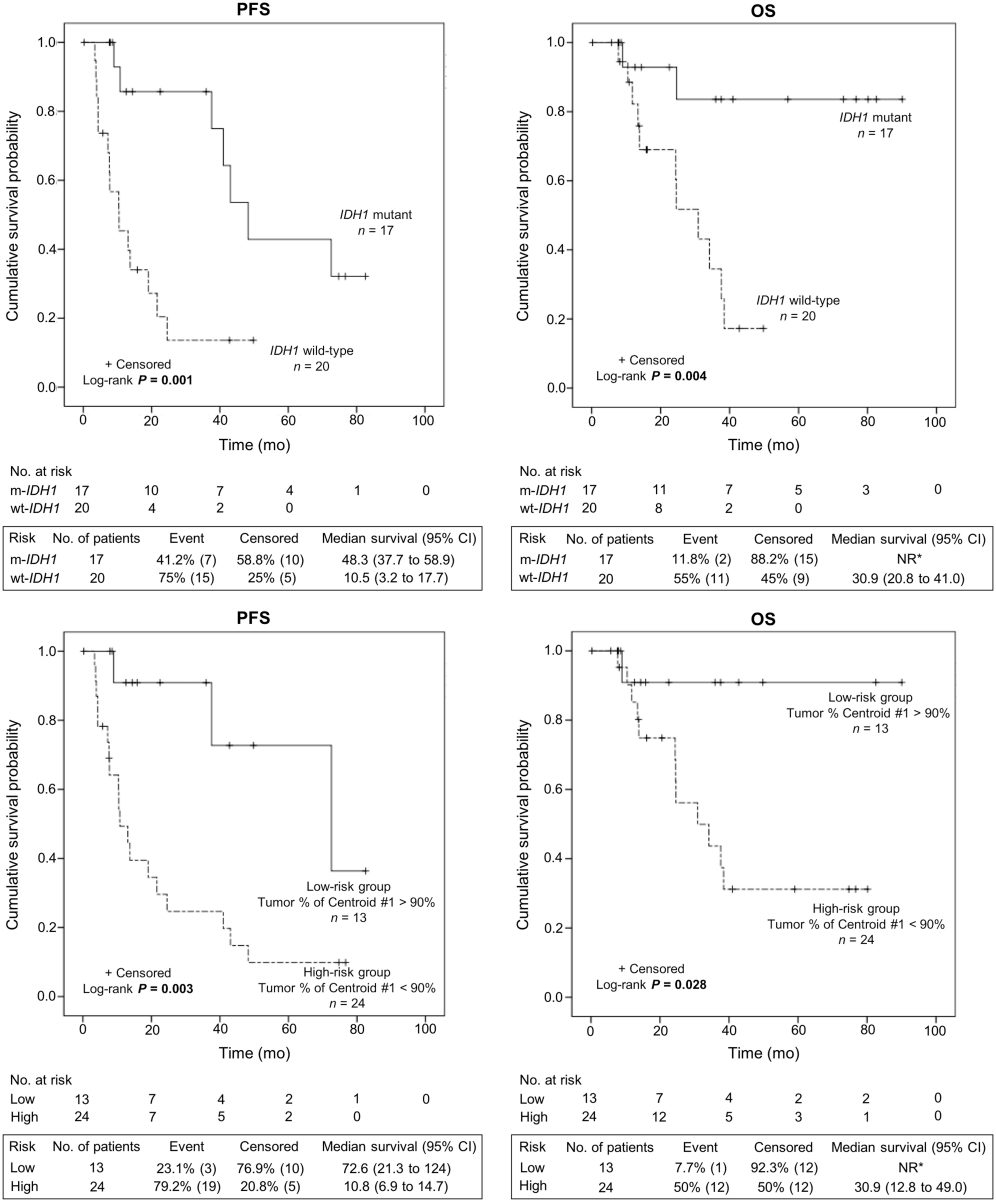
Kaplan-Meier estimates of progression-free survival (PFS) and overall survival (OS) according to IDH1 mutation (upper panel) status and tumor percentage of centroid #1 (lower panel). * NR = Not reached due to the lack of event.

**Fig 6:**
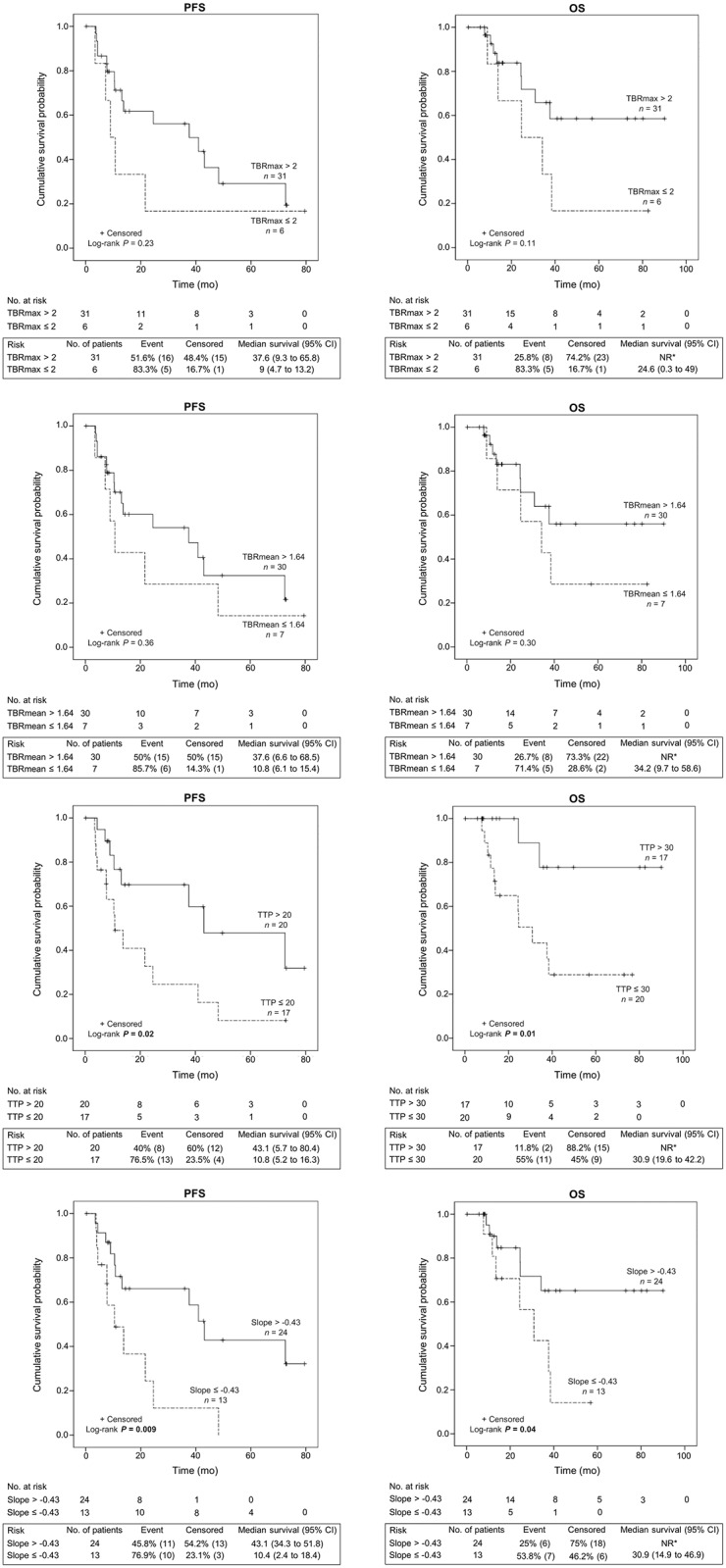
Kaplan-Meier estimates of progression-free survival (PFS) and overall survival (OS) according to other conventional ^18^F-FET PET parameters (TBR_max_, TBR_mean_, TTP, and slope). * NR = Not reached due to the lack of event.

The median (25^th^-75^th^ interquartile range) duration of follow-up was 22.5 months (11.8–38.4 months). Relapse/progression occurred in 22 patients (59%) at a median of 13.1 months (7.6–37.6 months) and 13 patients (35%) died from tumor progression. As shown in Fig 5, Kaplan-Meier estimates revealed that patients with IDH1 mutant tumors had a significant longer PFS (P = 0.001) and OS (P = 0.004) than IDH1 wild-type. Also, using X-tile software, a single optimal threshold was defined and showed that patients with a higher percentage of tumor voxel with centroid #1 > 90% had a longer PFS (P = 0.003) and OS (P = 0.028) due to the higher number of IDH1 mutant tumors (77% vs. 29% in patients with tumor voxel percentage of centroid #1 < 90%, P = 0.005). This remained significant after stratification on IDH1 mutation status (P = 0.029 for PFS and P = 0.034 for OS). Compared to other conventional ^18^F-FET PET parameters, only TTP and slope were associated with PFS and OS (P range 0.009 to 0.04, Fig 6).

## Discussion

Using an automatic clustering of TAC, we aimed at investigating the association between dynamic ^18^F-FET PET findings of whole-tumor voxels, IDH1 mutation status and survival in patients with gliomas. This kind of approach already has been proposed for MRI or ^18^F-FDG PET [35], however to the best of our knowledge, this is the first time it has been used for ^18^F-FET PET. This method can be easily applied for clinical routine. A trained physician can perform all the necessary steps from spatial normalization to segmentation in less than 5 minutes. The automatic TAC clustering, once it is firstly trained (to get the main centroids), is a matter of seconds. This method, as doesn’t involve physicians, is less subjective to bias compared to visual analysis, appears more reproducible and becomes integrated perfectly into the new area of large database. Also, as data were normalized, this kind of approach is more homogeneous between different centers and allows more feasible multicenter clinical trials.

Advantages of voxel-wise analysis of TAC include more precise characterization of tumor phenotypes and more precise assessment of survival. Indeed, when considering the whole tumor ROI for extracting the TAC, in 34 patients out of 37, the TAC’s shape of the whole tumor was in accordance with the predominant TAC (the one with the highest proportion). The whole tumor TAC shape didn’t reflect the majority of the voxels shape TAC in 3 patients. For example, in one patient with anaplastic astrocytoma IDH1 wild-type (patient #37), the extracted TAC was centroid #2 when considering a ROI on the whole tumor. When considering the proportion of each centroid, his prognosis was more defined. With a predominant centroid # 1 of 50.6%, the patient was classified as high-risk (as it was less than the threshold of 90%). Follow-up confirmed his worse survival with a low PFS and OS (3.4 and 13.8 months respectively). This discrepancy between both analyses may be explained by the fact that when a full ROI is taken, data are summed and some local variations of voxel of lower intensity may be lost. Then, retained information are the ones of the most actives voxels.

Shapes of TAC are not yet fully understood and are hypothesized to reflect many aspects of tumor microenvironment and tumor phenotype. Using PET MRI scan, Zhang et al. [23] reported that a positive correlation (r = 0.53; P = 0.002) was found on 2D ROI between ^18^F-FET PET tumor-to-brain ratio and regional cerebral blood flow as measured using arterial spin labeling. Nevertheless, as shown with the Pearson product moment “r”, even if there might be a positive correlation, it remained weak and didn’t fully reflect the pathophysiological complexity of gliomas. Xiong et al. found that IDH mutant tumors had also a lower microvascular density [36]. These findings are in accordance with our study as a lower microvascular density could lead, all other things equal otherwise, to a lower perfusion and therefore to a lower uptake and slope of shape. Our IDH1 mutant tumors had a lower ^18^F-FET uptake and as they were composed of majority of centroid #1 (with the lowest initial slope) and less centroid #3 (high initial slope). The centroid #3 is classically described as a high wash-out curve which may reflect local tumor aggressiveness due to a higher perfusion and an increased metabolic turnover in tumor cells. Also, our results are in line with those observed by Thon et al. [20], as the ascending curves would correspond to our centroid #1 and had a better prognosis in term of PFS (85% 2-year survival for homogeneous increasing TAC against 51% for focal decreasing TAC and 28% for homogeneous decreasing TAC).

IDH1 mutant tumors had a lower ^18^F-FET uptake in our cohort (Table 3). IDH1 and IDH2 belong to the NADP^+^- dependent IDH isoforms which are found in the cytosol for IDH1 and mitochondria for IDH2. IDH1 and IDH2 produce NADPH by catalyzing the oxidative decarboxylation of isocitrate to α-oxoglutarate (OG) outside of the Krebs cycle. NADPH plays a substantial role in cellular control of oxidative damage. Loss of enzyme activity due to the dominant negative effect of IDH mutants leads to a new enzymatic activity transforming α-cetoglutarate into 2-hydroxyglutarate (2-HG). This leads to an inhibition of the 2-OG dependent enzymes and may act as an oncometabolite with alternative molecular pathways [37] that may influence amino acid tracer uptake in the tumor [15]. Controversial results have been described for the effect of IDH mutant on the hypoxia-inducible factor (HIF) pathway which is known to stimulate the expression of glucose transporter (GLUT) and LAT. Indeed, as ^18^F-FET is not metabolized nor stocked, its signal only depends on the expression of the LAT and the intra/extracellular concentration of ^18^F-FET. IDH1 appears to function as a tumor suppressor that, when mutationally inactivated, contributes to tumorigenesis in part through the induction of the HIF-1 pathway. Zhao et al. reported that overexpression of IDH mutant in U87M glioma cells increased HIF-1α target expression proteins [38]. Nevertheless, another study showed that R-2HG that would promote the activation of EGLN1-2 or 3, which would result in the degradation of HIF-1α. Analysis of the gene expression from the TCGA data archives revealed that tumors expressing the IDH mutant had a reduced expression of HIF target genes compared to tumors containing IDH wild-type [39]. HIF is known to be related to the metabolism enzyme, particularly GLUT, but also LAT and even if its role remain unclear, it have been described in other cancers such as mesotheliomas or lung cancer [40]. Other amino acid tracers have been investigated. Recently, in 109 patients with gliomas, Lopci et al. showed that ^11^C-MET PET parameters were significantly correlated with histological grade and IDH1 mutation status. In this cohort, even if it didn’t reach the significance level (P = 0.05), SUV_max_ seemed to be inversely correlated with the presence of IDH1 mutation in this cohort [41]. Also, Verger et al. [42] found that IDH mutations were paradoxically correlated with a higher ^18^F-FDOPA uptake in diffuse gliomas. The apparent discrepancy between the uptake pattern of ^18^F-FET, ^11^C-MET and ^18^F-FDOPA may be linked to the metabolomic profile of IDH mutant tumors [15].

The current study had several limitations. The main limitation was the retrospective nature of the data collection that may have introduced a selection bias. Secondly, as some ^18^F-FET PET were visually normal and confirmed with normal Z-score maps, it was impossible to delineate 15 patients out of the 52 patients initially included. This may also have included a selection bias. Thirdly, we may criticize the generation of the “normal FET population” using 41 patients with visually normal ^18^F-FET PET and untreated gliomas. Indeed, those patients had abnormal MRI but which didn’t show any metabolic rendering. Nevertheless, using SPM, it was checked that each patient didn’t statistically differ from the 40 other patients and we do not think that it shall impact greatly on the final ROI because of the voxel that may be missed would be the ones with the smallest intensity value. Fourthly, glioblastomas (5 patients) which are classically IDH wild-type (90% of glioblastomas) for primary subtype, are highly metabolic tumors with a high amino acid metabolism and are associated with a poorer prognosis. Nevertheless, removing them didn’t change the significance level of our findings in an additional analysis. Two different PET/CT scanners were used in this study. It has been verified that the mean activity was not statistically different between both machines. After preprocessing, the resulting voxel were downsampled to the same size. All data were normalized from mean which shouldn’t impact the shape and tumor proportion of TAC. As no normalization was performed on the unnormalized data to compare the mean tumor activity between IDH1 wild-type and IDH1 mutant, it has been checked (data not shown) that activity was not statistically different between both PET/CT scanners. Also, the proportion of IDH1 mutant tumors was split equally between both PET/CT machines.

## Conclusion

In conclusion, based on dynamic ^18^F-FET PET acquisition, we developed a full automatic clustering approach of TAC which appears to be a valuable noninvasive diagnostic (for IDH1 mutant status) and prognostic marker in patients with gliomas. Further larger prospective studies are warranted to validate these findings.

## Supporting information

S1 Appendix Dynamic time warping (DTW). DTW aims to find a non-linear agreement between two-time series. Let’s consider two time series Q and C with the same number of time points n where Q = q1, q2…, qn and C = c1, c2 …, cn, it can build the M matrix of dimension n×n matrix whose i, j^th^ element is the Euclidean distance between qi and cj. Therefore, objective of DTW aims to find the path through M that minimizes the cumulative distance. The optimal path is found following recursive function: γ(i,j) = d(qi,cj) + min(γ(i−1,j−1), γ(i−1,j), γ(i,j−1)).
(DOCX)
