# Supplementary material for: Voxel-based 18F-FET PET segmentation and automatic clustering of tumor voxels: A significant association with IDH1 mutation status and survival in patients with gliomas
Source: PLoS One. 2018 Jun 28;13(6):e0199379. doi: 10.1371/journal.pone.0199379 (PMC6023198; doi:10.1371/journal.pone.0199379)
Supplement: S1 Appendix — DTW aims to find a non-linear agreement between two-time series. Let’s consider two time series Q and C with the same number of time points n where Q = q1, q2…, qn and C = c1, c2 …, cn, it can build the M matrix of dimension n×n matrix whose i, jth element is the Euclidean distance between qi and cj. Therefore, objective of DTW aims to find the path through M that minimizes the cumulative distance. The optimal path is found following recursive function: γ(i,j) = d(qi,cj) + min(γ(i−1,j−1), γ(i−1,j), γ(i,j−1)). (DOCX) [file pone.0199379.s001.docx]

**S1 Appendix. Dynamic time warping (DTW).** DTW aims to find a non-linear agreement between two-time series. Let’s consider two time series Q and C with the same number of time points n where Q = q1, q2…, qn and C = c1, c2..., cn, it can build the M matrix of dimension n×n matrix whose i, j^th^ element is the Euclidean distance between qi and cj. Therefore, objective of DTW aims to find the path through M that minimizes the cumulative distance. The optimal path is found following recursive function: γ(i,j) = d(qi,cj) + min(γ(i−1,j−1), γ(i−1,j), γ(i,j−1)).
